# Analysis of Amino Acid and Derivative Diversity and Antioxidant Capacity in *Ophiocordyceps sinensis* and Its Substitutes

**DOI:** 10.3390/jof11100711

**Published:** 2025-09-30

**Authors:** Haoxu Tang, Bing Jia, Chuyu Tang, Chao Feng, Yuling Li, Xiuzhang Li

**Affiliations:** State Key Laboratory of Plateau Ecology and Agriculture, Qinghai Academy of Animal and Veterinary Science, Qinghai University, Xining 810016, China

**Keywords:** *Ophiocordyceps sinensis*, amino acids, antioxidant activity, targeted metabolomics, multivariate statistical analysis

## Abstract

In this study, we used liquid chromatography–tandem mass spectrometry (LC-MS/MS) combined with multivariate statistical analysis to conduct comprehensive qualitative and quantitative profiling of amino acids and their derivatives in wild *Ophiocordyceps sinensis* (*O. sinensis*) samples from Naqu (NQ) and Xiaojin (XJ), cultivated *O. sinensis* (RG), and Bailing Capsules (BL). The objective was to systematically characterize amino acid metabolism and assess its correlation with antioxidant functionality. A total of 82 amino acids and their derivatives were identified. XJ had the highest essential amino acids, while BL had significantly lower content (except lysine) (*p* < 0.05). Antioxidant assays revealed that NQ and XJ samples exhibited superior antioxidant activity in 2,2′-azino-bis (3-ethylbenzothiazoline-6-sulfonate) (ABTS), 2,2-diphenyl-1-picrylhydrazyl (DPPH), and ferric ion reducing antioxidant power (FRAP) assays, and this activity showed a correlation to the contents of bioactive components such as total phenols (TPS), total polysaccharide (TPE), and total flavonoids (TF). Further pathway analysis using the Kyoto Encyclopedia of Genes and Genomes (KEGG) suggested that arginine and proline metabolism, aromatic amino acid biosynthesis, and tryptophan metabolism may be critical pathways that could underpin regional differences in *O. sinensis* quality, while variation in tyrosine metabolism may account for differences in antioxidant activity. This study provides a systematic comparison of amino acid profiles and antioxidant capacities across *O. sinensis* and its substitutes, offering a robust theoretical foundation for the development and functional evaluation of these bioresources.

## 1. Introduction

*Ophiocordyceps sinensis* (*O. sinensis*) is a parasitic fungus native to the high-altitude regions of the Qinghai–Tibet Plateau. Multiple studies have shown that it contains various active ingredients such as proteins, nucleotides, polyphenols, ergosterol, and polysaccharides [1,2,3,4]. It is well-recognized for its diverse pharmacological properties, encompassing antioxidant, immunomodulatory, anti-fatigue, and anti-inflammatory activities [1,2,5]. Due to its specialized ecological niche, complex life cycle, and increasing global demand, wild *O. sinensis* has become critically scarce. This scarcity has driven efforts toward artificial cultivation and the development of alternative fungal resources. Two main strategies have emerged, namely establishing controlled cultivation systems for *O. sinensis* and identifying alternative fungi with similar morphology and partial pharmacological properties such as *Cordyceps militaris* and *Isaria cicadae* [6,7,8]. Although these substitutes may resemble wild *O. sinensis* in appearance and share certain compounds, their metabolic profiles may differ substantially. Wild *O. sinensis* synthesizes specialized metabolites under extreme alpine conditions (low temperatures, high UV, unique microbiota) via complex regulatory networks, whereas controlled cultivation of substitutes and artificial products eliminates such stimuli [9]. Substitutes exhibit subtle genetic variations from selection/mutations [10,11]; artificial *O. sinensis* often involves genetic modification or clonal propagation, with both perturbing enzymatic activity and altering metabolite accumulation [12,13]. Wild *O. sinensis* undergoes dynamic metabolic shifts across its complex lifecycle, but premature harvest of substitutes and stage-skipping in artificial products cause deficits in stage-specific compounds. Additionally, symbiotic microbes enhance metabolite synthesis in wild *O. sinensis*, whereas simplified microbial environments for substitutes and artificial products reduce such synergies, exacerbating metabolic differences [14]. These factors collectively generate distinct metabolic fingerprints, affecting bioactivity, toxicology, and regulatory categorization. Among the bioactive components of *O. sinensis*, amino acids, as key primary metabolites, play crucial roles in both nutritional value and functional properties, thereby contributing significantly to the intrinsic quality of *O. sinensis* [15,16], and are linked to human health by aiding in disease prevention, including cancer [17,18]. Deficiencies in amino acids may impair protein synthesis and physiological functions, leading to symptoms such as fatigue, memory loss, and edema [19,20]. Beyond structural roles, amino acids serve as precursors for neurotransmitters, antioxidants, and signaling molecules [21]. For example, sulfur-containing amino acids (e.g., methionine, cysteine) are precursors of glutathione, and amino acids such as arginine and branched-chain amino acids (BCAAs) enhance immune responses and stress resistance [22,23]. Recent five-year studies have further confirmed that these sulfur-containing amino acids, as key precursors for glutathione synthesis, play a central role in regulating the oxidative stress balance in organisms [24,25]. Specifically, methionine can be converted into cysteine through the transsulfuration pathway, and cysteine, as the rate-limiting substrate for glutathione synthesis, directly determines the production level of glutathione, thereby influencing the organism’s ability to scavenge reactive oxygen species (ROS) and resist oxidative damage [26]. For *O. sinensis*, the exertion of its antioxidant function not only relies on classic bioactive components such as polysaccharides and phenols but also the significance of sulfur-containing amino acids as antioxidant precursor substances is gradually becoming prominent [27,28]. Therefore, variations in amino acid composition across wild, cultivated, and substitute samples may significantly affect their biological efficacy.

Antioxidant activity, which refers to the ability to neutralize ROS and free radicals, serves as a crucial parameter for evaluating the functional potential of natural products [29,30]. ROS can damage lipids, proteins, and DNA, triggering oxidative stress that contributes to chronic diseases such as cardiovascular and neurodegenerative disorders, diabetes, and cancer [29,30]. Antioxidant compounds help delay cellular aging and maintain physiological homeostasis through radical scavenging, metal ion chelation, and inhibition of lipid peroxidation [31,32]. As a medicinal fungus with high therapeutic value, the antioxidant potential of *O. sinensis* has attracted increasing attention. Previous studies have primarily focused on the nutritional quality comparison between wild and artificially cultivated *O. sinensis*, as well as investigations into the bioactive mechanisms of *O. sinensis* and its fermented mycelia, among other related areas [16,33,34]. Although metabolomics has enabled new insights into its biochemical landscape, relatively little is known about how amino acid composition varies among wild, cultivated, and substitute samples, or how these differences influence antioxidant activity. This knowledge gap impedes the accurate assessment of quality, authentication of resources, and standardization of *O. sinensis* products.

To facilitate more targeted development of *O. sinensis*, it is imperative to leverage metabolomics approaches for the identification and characterization of its key bioactive constituents. Targeted metabolomics, particularly liquid chromatography–tandem mass spectrometry (LC-MS/MS)-based amino acid profiling, offers a powerful tool for distinguishing samples by species and production method [3]. Understanding the relationship between amino acid metabolism and antioxidant performance is critical for evaluating functional equivalence, tracing origin, and guiding industrial application [35,36]. This study aimed to identify key differential amino acid metabolites (DAAMs) and their associations with antioxidant activities among wild, artificially cultivated *O. sinensis* and its substitutes using metabolomics, thereby providing a scientific basis for targeted development and quality evaluation of *O. sinensis* resources.

## 2. Materials and Methods

### 2.1. Sample Collection

HPLC-grade acetonitrile and methanol were purchased from Merck (Darmstadt, Germany), and Milli-Q water was obtained from Millipore (Bradford, PA, USA). Analytical-grade ammonium acetate and formic acid were sourced from Sigma-Aldrich (St. Louis, MO, USA). Stock solutions of 94 amino acid standards (Table S1) were stored at −20 °C.

Wild *O. sinensis* samples from Naqu (NQ; 91°39′13″ E, 31°7′16″ N; H: 4485 m) and Xiaojin (XJ; 102°32′13″ E, 30°28′16″ N; H: 3060 m) were collected in June 2024 from Qinghai Baohuitang Biotechnology Co., Ltd. (Xining, China). Artificial *O. sinensis* (RG) was obtained from Sunshine Lake Pharma Co., Ltd. (Dongguan, China) and Bailing Capsules (BL) samples were sourced from Huadong Medicine Co., Ltd. (Hangzhou, China). All fresh samples were stored at −86 °C until further metabolomic and antioxidant analysis.

### 2.2. Metabolite Extraction

A 50 ± 2.5 mg portion of the *O. sinensis* sample was placed into a 2 mL centrifuge tube. Next, 500 µL of 70% methanol aqueous solution, pre-cooled to −20 °C and purchased from Merck KGaA (Darmstadt, Germany), was added to the tube. The mixture was shaken for 3 min, after which it was centrifuged at 12,000 rpm for 10 min under 4 °C conditions. A 300 µL aliquot of the supernatant was then transferred to a 1.5 mL tube, which was subsequently stored at −20 °C for 30 min to allow standing.

### 2.3. LC-MS/MS Analysis

The analysis was performed using established protocols. We employed an UltraPerformance Liquid Chromatography (UPLC) ExionLC™ AD system (SCIEX, Framingham, MA, USA; https://sciex.com.cn/, accessed on 10 October 2024) and a Tandem Mass Spectrometer QTRAP^®^6500+ (SCIEX, Framingham, MA, USA; https://sciex.com.cn/ accessed on 25 October 2024) for sample analysis. The separation was achieved using ACQUITY BEH Amide columns (1.7 µm, 100 mm × 2.1 mm i.d., Waters Corporation, Milford, MA, USA), with a detection threshold of 0.05 ng/mL. Operating conditions were set with a column temperature at 40 °C, an injection volume of 2 µL, and a flow rate of 0.4 mL/min. The mobile phase consisted of two components: phase A contained ultrapure water with 2 mM ammonium acetate and 0.04% formic acid, while phase B comprised acetonitrile with 2 mM ammonium acetate and 0.04% formic acid. The gradient elution started at a 10:90 (*v*/*v*) A/B ratio for the initial 1.2 min, shifted to a 40:60 (*v*/*v*) ratio over 9 min, adjusted to a 60:40 (*v*/*v*) ratio between min 10 and 11, and concluded by returning to a 10:90 (*v*/*v*) ratio from 11.01 to 15 min. For mass spectrometry, the electrospray ionization (ESI) was set at 550 °C, with a voltage of 5500 V for positive mode and −4500 V for negative mode. The Curtain Gas (CUR) pressure was set at 35 psi. The Q-Trap 6500^+^ system scanned and detected ion pairs using optimized declustering potential (DP) and collision energy (CE) settings. Quantitative analysis was conducted using triple quadrupole mass spectrometry in the Multiple Reaction Monitoring (MRM) mode. Post-data acquisition, chromatographic peaks were integrated and analyzed against a standard curve. A Metware V 1.0 Database (MWDB) was compiled to assist in interpreting the mass spectrometry data. Quality control (QC) samples were prepared from pooled *O. sinensis* samples to maintain analytical consistency, introduced after every set of 10 samples.

### 2.4. Data Processing

The raw LC-MS/MS data were processed using the MultiQuant 3.0.3 software. Data matrices comprising retention time, mass-to-charge ratio, and peak intensity were constructed after filtering out peaks with a relative standard deviation (RSD) exceeding 30% in the quality control (QC) samples. In LC-MS analysis, the retention times of metabolites, along with the precursor and fragment ions in Multiple Reaction Monitoring (MRM) mode, were determined from metabolite standards, facilitating the identification and quantification of metabolites in the samples. Additionally, the mass spectrometry data were aligned with public metabolic databases, including the Human Metabolome Database (HMDB) (http://www.hmdb.ca/, accessed on 11 October 2024) and Metlin (https://metlin.scripps.edu/, accessed on 11 October 2024), as well as MeiJi’s custom libraries, to gather comprehensive information on metabolites. Peaks with more than 50% missing values across the group were excluded from further statistical analysis to maintain analytical rigor. The data underwent normalization by total peak area and were transformed using Log10 scaling. Python version 3.12 was employed for pattern recognition, and the data were preprocessed with unit variance scaling (UV) to simplify subsequent analysis. Principal Component Analysis (PCA) was conducted to discern overall metabolic differences and sample variations. Hierarchical Cluster Analysis (HCA) was utilized to explore metabolite accumulation patterns under various conditions, visualized through heatmaps with corresponding dendrograms. DAAMs were selected based on fold change ≥ 2 and fold change ≤ 0.5.

### 2.5. Calibration Curves Quantification

In this study, a series of standard solutions with varying concentrations (10, 20, 50, 100, 200, 500, 1000, 2000, 5000, 10,000, and 20,000 ng/mL) were prepared, and mass spectrometry peak intensity data were collected at each concentration. During the experiment, the *x*-axis represents the concentration ratio between the external standard and the internal standard, while the *y*-axis indicates the peak area ratio between the external standard and the internal standard. By employing this method, standard curves for different compounds were plotted, and a quantitative model for 94 amino acid metabolites was established based on these curves. The quantification of amino acid metabolites involved substituting the ratio of the integrated peak area from the identified samples into the linear equation of the standard curve for computation. This equation was then applied in a computational method to determine the substance content data (Y) in the actual samples (Table S2).

### 2.6. Measurement of Physiological and Biochemical Parameters

The physiological and biochemical properties of *O. sinensis* are categorized into functional components and antioxidant active components. Functional components include total polysaccharide (TPE), total phenols (TPS), total flavonoids (TF), total anthocyanins (TA), chlorophyll (CH), proanthocyanidins (PC), mannitol, crude proteins, and adenosine. Antioxidant activity was determined using 2,2′-azino-bis (3-ethylbenzothiazoline-6-sulfonate) (ABTS), ferric ion reducing antioxidant power FRAP, and 2,2-diphenyl-1-picrylhydrazyl (DPPH) assays. These 12 physiological and biochemical indices were determined using detection kits, following the instructions provided with the kits. All kits were purchased from Suzhou Keming Biotechnology Co., Ltd. (Suzhou, China).

## 3. Results

### 3.1. Physiological and Biochemical Characteristics of Four O. sinensis Samples

**Figure 1:**
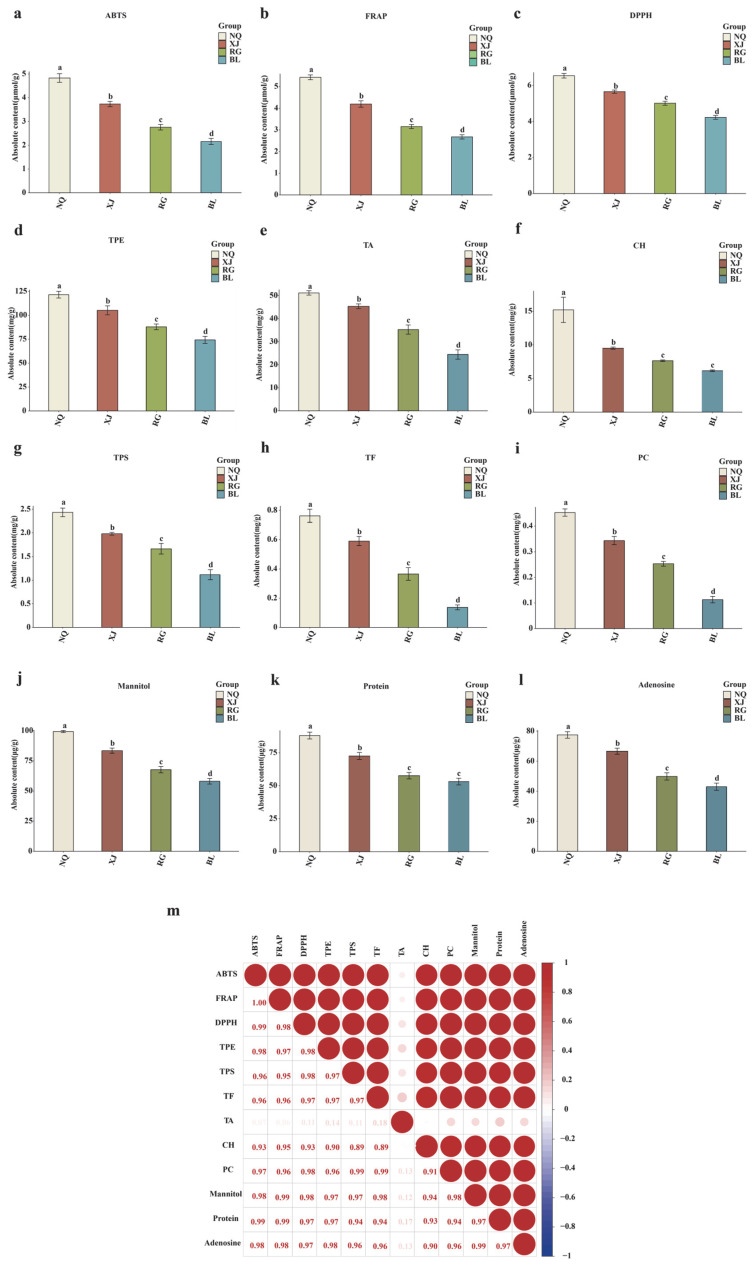
Comparison of antioxidant activities and key indicator components among different samples. (**a**) ABTS; (**b**) FRAP; (**c**) DPPH; (**d**) TPE content; (**e**) TA content; (**f**) CH content; (**g**) TPS content; (**h**) TF content; (**i**) PC content; (**j**) Mannitol content; (**k**) Protein content; (**l**) Adenosine content; (**m**) Heatmap showing the correlation between antioxidant activities and key indicator components. Distinct lowercase letters indicate significant differences in amino acid contents among the samples (*p* < 0.05), and the order of the letters represents the relationship between the mean values of metabolite contents.

We systematically assessed the antioxidant capacity and levels of bioactive compounds in *O. sinensis* from four sources, namely, NQ, XJ, RG, and BL (Figure 1a). In terms of antioxidant capacity, the three indicators of ABTS, FRAP, and DPPH in the NQ and XJ groups were significantly higher than those in the RG and BL groups (*p* < 0.05). Among them, the NQ group exhibited the strongest performance, followed by the XJ, RG, and BL groups in that order. NQ and XJ exhibited significantly higher contents of TPE, TPS, TF, TA, CH, PC, mannitol, protein, and adenosine compared to RG and BL (*p* < 0.05).

All measured components showed statistically significant differences across groups (*p* < 0.05), with the exceptions of protein and CH, which did not differ significantly between the RG and BL groups (*p* > 0.05). Specifically, the protein content was 57.62 μg/g in the RG group versus 53.16 μg/g in the BL group. Meanwhile, the CH content was 7.65 mg/g in the RG group compared with 6.16 mg/g in the BL group.

To explore the relationship between functional metabolites and antioxidant performance, a correlation heatmap (Figure 1m) was constructed. Among the findings, a potential strong positive correlation between functional ingredients and antioxidant content suggests that the accumulation of functional metabolites might play a key role in enhancing antioxidant capacity in *O. sinensis*. It is possible that key indicator components and antioxidant functions could exhibit positive or negative correlations, which would further reflect the complex relationship between metabolic components and biological activities of *O. sinensis*.

### 3.2. Amino Acid Metabolic Profiling

**Figure 2:**
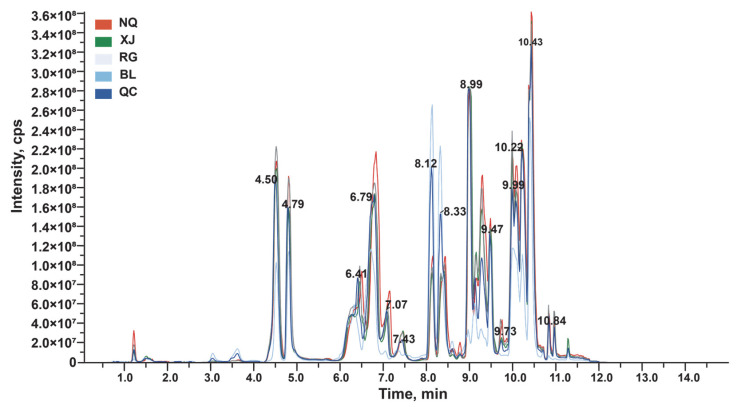
TIC of QC, NQ, XJ, RG and BL.

To explore the underlying causes of these differences, the present study performed targeted metabolomic analysis of amino acids and their derivatives, which are key primary metabolites closely linked to nutritional value and functional properties, using LC-MS/MS to achieve comprehensive qualitative and quantitative profiling. This analysis successfully quantified 82 amino acids in *O. sinensis*. The total ion chromatograms (TIC) for both the quality control (QC) samples showed high consistency and well-aligned retention times, indicating the presence of a wide range of metabolites and demonstrating the robustness of the analytical platform (Figure 2).

**Figure 3:**
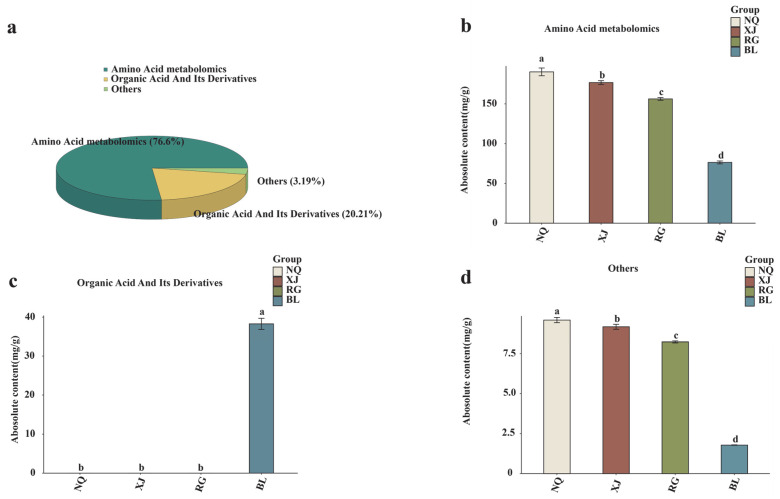
(**a**) Pie-chart classification of the 82 detected metabolites into three categories (Amino acid metabolomics, Organic acid and its derivatives, and others) in NQ, XJ, RG, and BL samples, showing their respective proportions. (**b**) The quantitative expression levels of Amino Acid metabolomics across NQ, XJ, RG, and BL sample types. (**c**) The absolute content of Organic acid and its derivatives in NQ, XJ, RG, and BL samples. (**d**) The quantitative expression levels of the “Others” metabolite category across NQ, XJ, RG, and BL sample types. Distinct lowercase letters indicate significant differences in amino acid contents among the samples (*p* < 0.05), and the order of the letters represents the relationship between the mean values of metabolite contents.

To comprehensively delineate the metabolic characteristics of *O. sinensis* and its substitutes, targeted metabolomic profiling was conducted using LC-MS/MS. A total of 82 amino acids and their derivatives were annotated across the groups. Among them, amino acid metabolomics was predominant, with 72 metabolites (76.6%), followed by organic acids and their derivatives, accounting for 7 metabolites (20.21%), and a small fraction of miscellaneous compounds, consisting of 3 metabolites (3.19%) (Figure 3a). Quantitative comparison among the groups revealed a distinct metabolic hierarchy. In terms of amino acid metabolomics and other metabolites, NQ exhibited the highest content, followed by XJ, RG, and BL in descending order. Regarding the content of organic acids and their derivatives, BL showed the highest level at 38.27 mg/g. Notably, the contents of organic acids and their derivatives in NQ, XJ, and RG were all zero. (Figure 3b–d). Among the samples from NQ and XJ regions, the most abundant amino acids and their derivatives are γ-aminobutyric acid, argininosuccinic acid, and succinic acid. In the RG samples, creatine phosphate, L-glutamic acid, and succinic acid are the predominant ones. Meanwhile, in the BL samples, aminobutyric acid, argininosuccinic acid, and succinic acid have the highest contents. Collectively, these results demonstrate that wild *O. sinensis*, its cultivated varieties, and substitutes exhibit distinct differences in both the composition and content of amino acids and their derivatives. Collectively, these results demonstrate that wild *O. sinensis*, its cultivated varieties, and substitutes exhibit distinct differences in both the composition and content of amino acids and their derivatives. Through targeted amino acid metabolomic profiling, this study provides critical insights into unraveling the characteristics of amino acid compositions and related components that distinguish these samples.

### 3.3. Multivariate Statistical Analysis of Amino Acid Profiles

**Figure 4:**
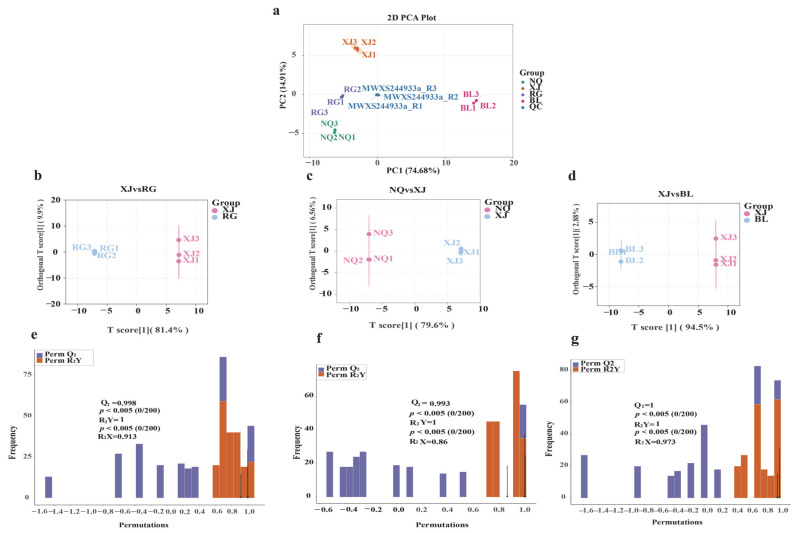
Multivariate Analysis of Amino Acid Metabolites in NQ, XJ, RG, and BL (**a**) PCA of samples from NQ, XJ, RG, BL, and QC. (**b**–**d**) OPLS-DA score plots for NQ, XJ, RG, and BL. (**e**–**g**) OPLS-DA permutation test results for NQ, XJ, RG, and BL. The arrows represent the values of Q^2^, R^2^X, and R^2^Y respectively.

To evaluate the metabolic distinctions among groups and the consistency within each group, PCA was conducted on the amino acid metabolite dataset. A total of 15 samples were analyzed, comprising 12 *O. sinensis* samples (Figure 4a). The first two principal components explained 74.68% and 14.91% of the total variance, respectively. The PCA score plot showed tight clustering within each sample group and clear separation among groups, indicating pronounced metabolic differences among NQ, XJ, RG, and BL. The QC samples clustered near the center, suggesting minimal technical variation and demonstrating the robustness and reproducibility of the analytical procedure.

In contrast to PCA, orthogonal partial least squares discriminant analysis (OPLS-DA) is a supervised method that enhances classification and improves predictive performance. To clarify the specific amino acid metabolic differences between wild *O. sinensis* and its related varieties, artificial breeds, and substitutes, we focused on pairwise comparisons between XJ and NQ (representing wild *O. sinensis*) and the other groups (BL, RG). After removing QC samples, OPLS-DA was employed to better discriminate among the sample groups. In this model, cumulative R^2^X and R^2^Y represent the explained variance in the predictor (X) and response (Y) matrices, respectively, while Q^2^ reflects the predictive accuracy of the model. Pairwise OPLS-DA comparisons revealed distinct metabolic separations: BL vs. XJ (R^2^X = 0.973, R^2^Y = 1.000, Q^2^ = 1.000), RG vs. XJ (R^2^X = 0.913, R^2^Y = 1.000, Q^2^ = 0.998), and NQ vs. XJ (R^2^X = 0.860, R^2^Y = 1.000, Q^2^ = 0.993). All models demonstrated excellent statistical performance, confirming the presence of region-specific metabolomic signatures.

To assess model reliability and avoid overfitting, 200-permutation tests were performed for each comparison. All models yielded permutation-derived *p*-values < 0.05 for both Q^2^ and R^2^Y, supporting their statistical significance. Additionally, all R^2^X values exceeded 0.5, indicating substantial explanatory power (Figure 4e–g).

**Figure 5:**
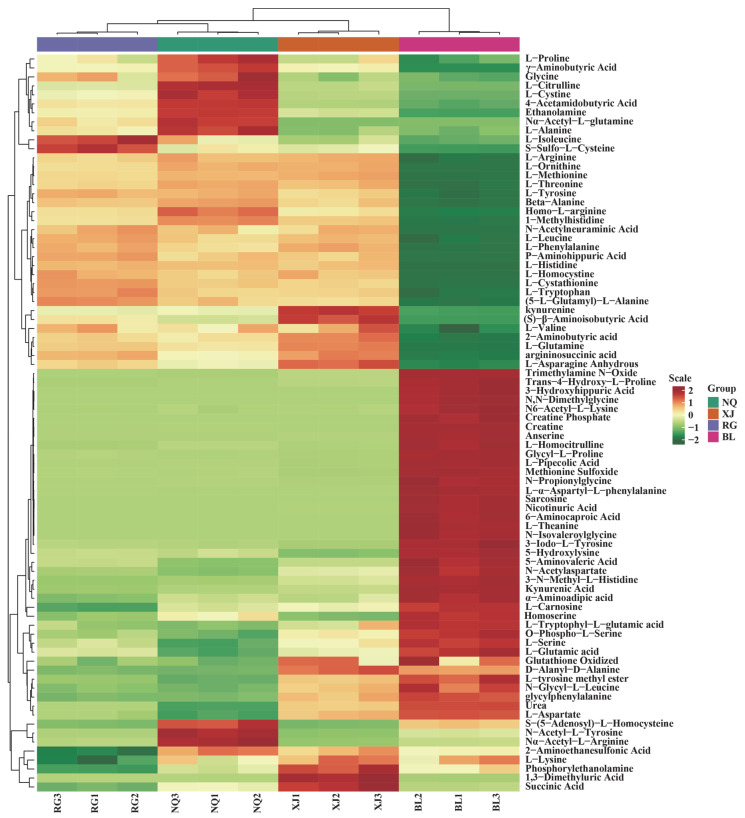
HCA of amino acid contents among *O. sinensis* samples is shown herein. The left side of the figure lists the names of each sample, while the right side provides a dendrogram that demonstrates the clustering status of these samples. The upper section of the figure presents the dendrogram for metabolite clustering, and the lower section displays the names of the metabolites. The proximity between sample branches reflects the similarity in the cumulative patterns of all metabolites between two samples. In Figure 5, red represents metabolites with higher expression levels in the respective sample, whereas green denotes those with lower expression levels.

HCA was performed on the Z-score-normalized peak area data of 82 amino acid metabolites from all sample types, and the results were visualized as a heatmap (Figure 5). The analysis identified two principal metabolite clusters, of which Cluster I, consisting of 35 metabolites predominantly enriched in NQ, XJ and RG samples, was characterized by proline and γ-aminobutyric acid as key representatives. In contrast, Cluster II included 47 metabolites, which showed higher abundance in BL samples, with glutamic acid, L-theanine and succinic acid as characteristic metabolites.

Group-specific metabolite distribution patterns were evident. Metabolites enriched in the upper region of the heatmap, such as proline and γ-aminobutyric acid, were predominantly accumulated in NQ, XJ and RG. Metabolites located in the central region of the heatmap, such as glutamic acid and L-theanine, were more abundant in BL, while those mapped to the lower section, including urea and L-aspartate, were mainly concentrated in BL. These results indicate pronounced metabolomic divergence among the four sample types, with BL exhibiting the most distinctive profile.

### 3.4. Identification and Characterization of DAAMs

**Figure 6:**
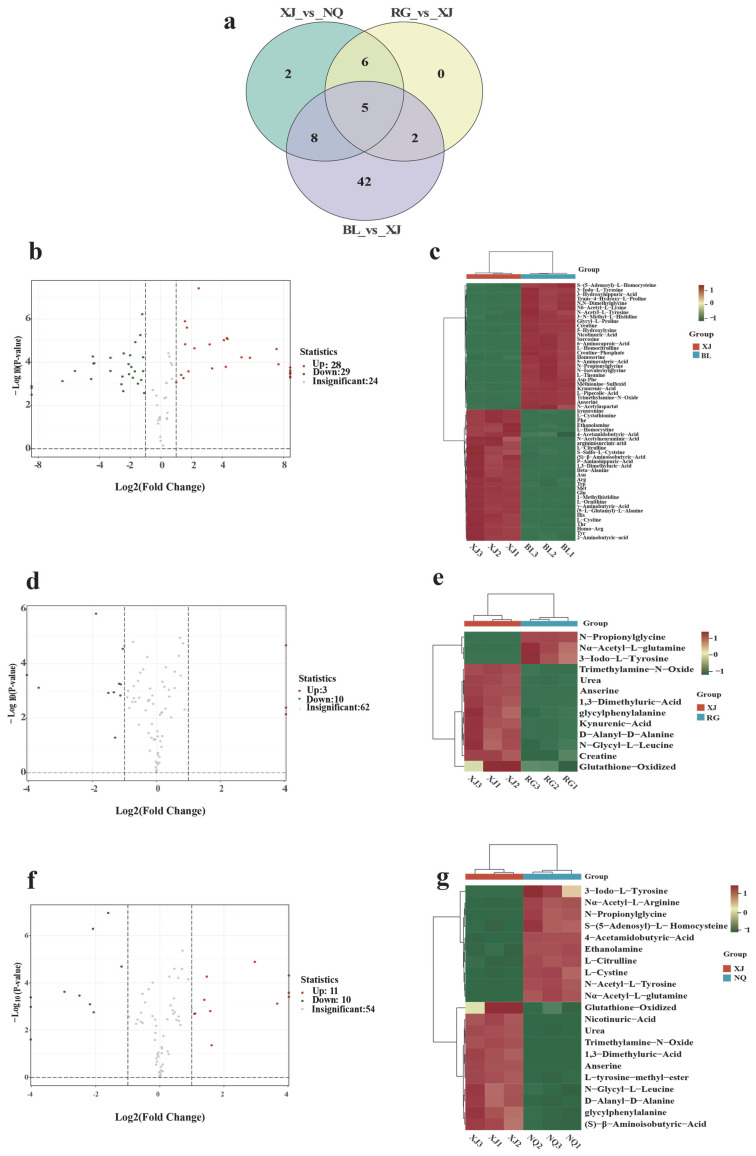
DAAMs of *O. sinensis* (**a**) Venn diagram of overlapping and unique metabolites among groups; (**b**) Volcano plot XJ vs. BL; (**c**) Hierarchical clustering XJ vs. BL; (**d**) Volcano plot XJ vs. RG; (**e**) Hierarchical clustering XJ vs. RG; (**f**) Volcano plot XJ vs. NQ; (**g**) Hierarchical clustering XJ vs. NQ.

To identify significantly altered amino acid metabolites and potential biomarkers among the sample groups. DAAMs were selected based on fold change ≥ 2 and fold change ≤ 0.5. DAAMs were visualized through volcano plots and Venn diagrams, and hierarchical clustering was conducted for each pairwise comparison to highlight distinct metabolic profiles (Figure 6c,e,g).

In total, 57 DAAMs between BL and XJ (28 upregulated, 29 downregulated; Figure 6b), 13 DAAMs between RG and XJ (3 upregulated, 10 downregulated; Figure 6d), and 21 DAAMs were identified between NQ and XJ (11 upregulated, 10 downregulated; Figure 6f). Among these DAAMs, the common DAAMs are 1,3-Dimethyluric-Acid, Trimethylamine-N-Oxide, 3-iodo-L-tyrosine, N-propionylglycine, and anserine (Figure 6a).

The comparative analysis of metabolic profiles reveals significant differences between XJ and BL, as evidenced by distinct expression patterns in the heatmap (Figure 6c). Notably, 1,3-Dimethyluric-Acid tends to show markedly higher levels in XJ, while trimethylamine-N-oxide and 3-iodo-L-tyrosine are likely more abundant in BL; the complete separation of these groups in hierarchical clustering may indicate potential fundamental differences in their metabolic composition. When comparing XJ to RG, metabolites like creatine and oxidized glutathione exhibit significant expression disparities (Figure 6e), though their closer clustering distance suggests some shared metabolic features between wild and cultivated varieties. Further regional specificity emerges in the XJ-NQ comparison (Figure 6g), where XJ demonstrates higher 1,3-Dimethyluric-Acid levels while NQ shows elevated S-(5′-Adenosyl)-L-homocysteine, potentially reflecting adaptations to different geographic conditions. These observed variations in metabolite expression profiles across sample groups may hold important implications for understanding their functional attributes. These variations can stem from different causes, including origin (wild/cultivated), environmental factors, production methods, host differences, and variations in associated microorganisms. However, direct correlations between such variations and antioxidant activity still require further validation through subsequent assays. The comprehensive metabolic fingerprinting presented herein establishes a foundation for future investigations, which will explore how these differences translate to functional properties like antioxidant capacity or nutritional quality.

**Figure 7:**
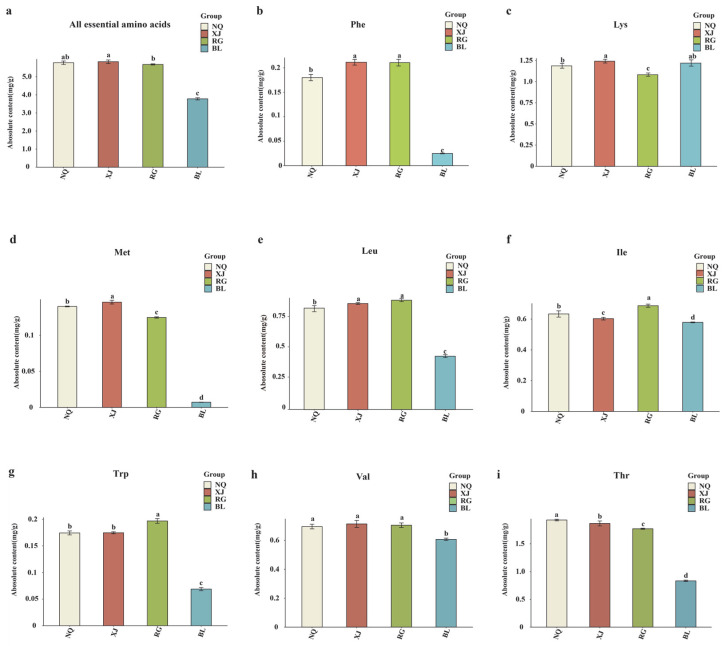
(**a**–**i**) Difference analysis of essential amino acid metabolites content. This study employs a one-way ANOVA to analyze essential amino acids. The horizontal axis denotes group names, while the vertical axis illustrates the metabolite contents of amino acids. Distinct lowercase letters indicate significant differences in amino acid contents among the samples (*p* < 0.05), and the order of the letters represents the relationship between the mean values of metabolite contents.

Amino acids are essential for maintaining physiological homeostasis in both humans and animals. Because many essential amino acids cannot be synthesized de novo, they must be obtained exogenously through dietary intake [37]. *O. sinensis* is a particularly rich source of these essential amino acids and has long been valued as a medicinal fungus in traditional Chinese medicine [38]. As essential amino acids serve as critical building blocks for protein biosynthesis and play pivotal roles in regulating various physiological processes, their quantitative characterization is of great significance for evaluating the nutritional quality of *O. sinensis* and its substitutes. Therefore, we further conducted quantitative analysis and visualization of the eight essential amino acids identified in the samples. In our comparative metabolomic profiling of four *O. sinensis* samples (NQ, XJ, RG, and BL), a total of 82 amino acid-related metabolites were identified, including eight essential amino acids: L-phenylalanine, L-threonine, L-tryptophan, L-lysine, L-methionine, L-isoleucine, L-leucine, and L-valine (Figure 7).

Among these samples, XJ exhibited the highest total content of essential amino acids at 5.84 mg/g, followed by NQ, RG, and BL (*p* < 0.05). This finding indicates that wild-harvested *O. sinensis* possesses a superior amino acid profile compared to artificially cultivated samples or substitutes. BL, as a substitute for *O. sinensis*, showed the lowest levels in seven essential amino acids except for lysine, with the lysine content in BL (1.24 mg/g) being higher than that in RG (1.08 mg/g) (*p* < 0.05). These amino acids were significantly more abundant in wild-harvested (NQ and XJ) and artificially cultivated (RG) samples. Although RG contained fewer essential amino acids than XJ and NQ, it had the highest levels of phenylalanine, leucine, isoleucine, tryptophan, and valine. Among XJ and NQ, significant differences were observed in all essential amino acids except for Trp and Val, where no significant difference was found (*p* < 0.05). Specifically, NQ contained higher levels of isoleucine and threonine compared to XJ, whereas XJ exhibited relatively higher contents of phenylalanine, lysine, methionine, and leucine (*p* < 0.05). These findings collectively highlight the profound impact of ecological origin and cultivation practices on the nutritional quality of *O. sinensis*, providing critical insights into the evaluation of wild resources and the optimization of artificial cultivation strategies for functional amino acid enrichment.

### 3.5. Correlation Between Amino Acids and Physiological-Biochemical Characteristics

**Figure 8:**
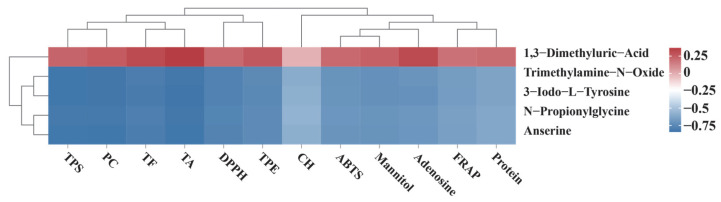
Intragroup Correlation Analysis of DAAMs.

To identify amino acids potentially influencing the physiological and biochemical traits of wild and cultivated *O. sinensis* as well as their commercial substitutes, five common DAAMs were selected for correlation analysis (Figure 8). These metabolites were correlated with three antioxidant indices (ABTS, FRAP, and DPPH) and nine functional components, including TPE, TPS, TF, TA, CH, PC, mannitol, protein, and adenosine. The resulting correlations were visualized in a heatmap. The analysis revealed that 1,3-Dimethyluric-Acid was positively correlated with all antioxidant activities and functional compounds. Conversely, Trimethylamine-N-Oxide, 3-iodo-L-tyrosine, N-propionylglycine, and anserine exhibited negative correlations with most indicators, with correlation coefficients frequently below −0.6. This negative association was particularly pronounced for functional components such as TPS.

### 3.6. KEGG Pathway Enrichment and Key Functional Annotations

Kyoto Encyclopedia of Genes and Genomes (KEGG) enrichment analysis across the three comparative groups identified multiple enriched metabolic pathways, which were broadly categorized into four functional classes genetic information processing, cellular processes, environmental information processing, and metabolism.

**Figure 9:**
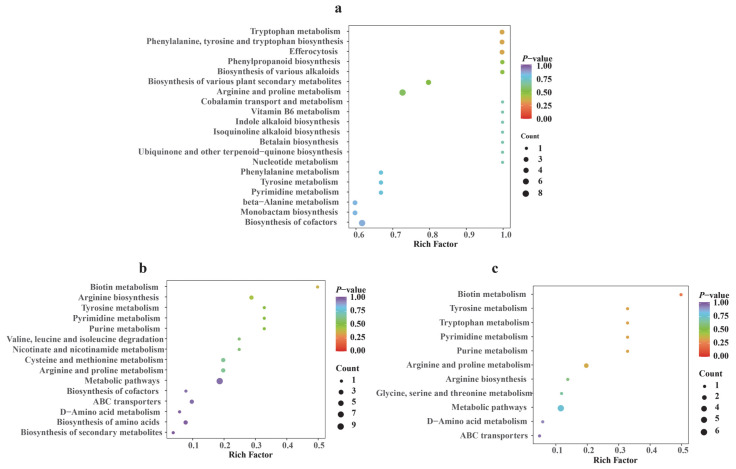
Pathway analysis of DAAMs. KEGG enrichment analysis of DAAMs between groups. The *x*-axis represents the enrichment score indicating the degree of enrichment, and the *y*-axis lists the enriched metabolic pathways. Larger bubbles represent a greater number of DAAMs involved in a pathway, while the color gradient from blue to red indicates decreasing *p*-values and increasing statistical significance. (**a**) BL vs. XJ, (**b**) RG vs. XJ, (**c**) NQ vs. XJ.

From the bubble plots, the commonly enriched pathways across comparisons include amino acid metabolism, which encompasses tyrosine metabolism (Figure 9a,c), arginine-proline metabolism (Figure 9a–c), and glycine, serine and threonine metabolism (Figure 9c). Secondary metabolite biosynthesis includes pathways such as Indole alkaloid biosynthesis (Figure 9a) and biosynthesis of secondary metabolites (Figure 9b). Nucleotide metabolism, involving purine and pyrimidine metabolism (recurrently enriched in plots a, b, and c), supports nucleic acid synthesis and energy turnover (e.g., ATP production). Core metabolism and transport-related pathways exhibit partial distribution across plots. Plot a lacks typical transport pathways (e.g., ABC transporters) and plot b does not explicitly display such pathways, but both contain components of basic metabolic networks; plot c, however, includes ABC transporters involved in transmembrane transport, and together these pathways underpin essential cellular functions, including the maintenance of metabolic networks and material exchange [39,40]. Notably, pathways such as tyrosine metabolism, pyrimidine metabolism, and arginine-proline metabolism were repeatedly enriched in multiple comparisons, and the accumulation of DAAMs associated with these pathways further highlights their potential biological significance in mediating metabolic phenotypes.

By integrating pathway enrichment results with heatmap analysis of amino acid metabolism, given that DAAMs are primarily enriched in pathways such as tyrosine metabolism, arginine and proline metabolism, and aromatic amino acid biosynthesis, these pathways are likely to be the key ones influencing their quality [41]. Notably, precursor compounds such as tryptophan, phenylalanine, and tyrosine were significantly elevated in XJ and NQ samples, indicating more active metabolic processes in these groups. Conversely, the XJ sample showed an upregulation trend in metabolites associated with the phagosome pathway, potentially reflecting enhanced cellular repair and homeostasis mechanisms in response to environmental stress [42].

Further KEGG enrichment analysis demonstrated that common DAAMs were enriched in general metabolic pathways and specifically in tyrosine metabolism. As a central pathway within aromatic amino acid metabolism, tyrosine metabolism is closely linked to antioxidant activity [21,43]. Metabolites involved in this pathway, such as tyrosine and its derivatives, may influence the antioxidant potential of *O. sinensis* by modulating redox reactions or serving as precursor molecules. The widespread enrichment of metabolic pathways underscores the importance of coordinated amino acid networks in maintaining antioxidant functions. Collectively, these findings elucidate potential molecular mechanisms underlying the antioxidant activity of *O. sinensis* and provide a theoretical basis for targeting key metabolic pathways to enhance its medicinal efficacy.

## 4. Discussion

Metabolomics, emerging after genomics and proteomics, employs high-throughput technologies to analyze small molecules (<1000 Da) in biological samples, aiming to link these metabolites to physiological processes and identify potential biomarkers through pathway enrichment analysis [44,45]. This approach is widely utilized in medicine [46,47], food science, and plant research [48].

In this study, targeted metabolomics was applied to quantitatively analyze amino acids and their derivatives in wild *O. sinensis*, cultivated, and substitute samples, revealing significant differences in amino acid and its derivatives content and metabolic pathways among the four types. *O. sinensis* exhibits notable pharmacological effects, many attributed to its antioxidant properties [49]. The overall superior performance of wild *O. sinensis* (NQ and XJ) in both antioxidant capacity and essential amino acid accumulation compared to RG and BL may potentially stem from the synergistic effects of its harsh growth environment, specific host-insect association, and symbiotic microbial interactions. RG and BL may either lack these key drivers or have disrupted them, which could result in wild *O. sinensis* potentially exhibiting the relatively highest levels in both traits, with RG possibly following as the intermediate, and BL may show the relatively lowest levels among the three. Wild *O. sinensis* is naturally exposed to the harsh high-altitude environments characterized by low temperatures, low oxygen availability, and increased ultraviolet (UV) radiation [50]. These extreme conditions likely stimulate adaptive responses that enhance the production of secondary metabolites (including antioxidants) and may also promote the synthesis of essential amino acids, two traits closely linked to its metabolic superiority. Antioxidants play a crucial role in protecting the organism from oxidative stress induced by these environmental factors [51]. Consequently, the prolonged exposure to such stressful conditions may drive the wild strains to develop more robust antioxidant mechanisms, contributing to superior growth and metabolic activity compared to their cultivated counterparts, which lack these environmental pressures [52,53]. Beyond environmental stimuli, wild *O. sinensis* forms a unique insect-fungus complex with its specific host caterpillar (e.g., larvae of *Hepialidae*), and this host association may be critical for metabolite synthesis [54]. The host might provide specialized nutrients (such as insect-derived amino acids and lipids) and regulatory signals that could trigger the expression of genes involved in antioxidant-related metabolism (e.g., pathways for phenols and polysaccharides synthesis) [55]. Additionally, symbiotic microbes (e.g., endophytic bacteria and fungi) in the wild habitat may further enhance metabolite accumulation as these microbes could secrete growth-promoting substances and co-metabolites that synergistically boost the production of antioxidants and their precursors (e.g., essential amino acids) in wild *O. sinensis* [56,57].

In contrast, RG is cultivated in a constant and controlled environment (stable temperature, regulated UV, and standardized nutrient supply) that lacks the extreme stressors of high-altitude habitats. This stable setting not only fails to activate stress-responsive metabolic pathways (e.g., those driving antioxidant synthesis) but also may lack the specific host-insect signals and symbiotic microbial networks of wild *O. sinensis* [54,58]. As a result, RG shows reduced accumulation of functional components (TPS, TPE, TF) and essential amino acids, which may lead to significantly lower antioxidant activity in ABTS, DPPH, and FRAP assays compared to wild samples. BL, as a commercial substitute, differs more fundamentally. It is typically composed of mycelia from alternative fungal species (not the natural *O. sinensis* insect–fungus complex) and produced via fermentation in a simplified microbial environment [59]. BL completely lacks the host-insect association of wild *O. sinensis*, which may deprive it of host-derived nutrients and regulatory cues; meanwhile, its simplified microbial community cannot provide the symbiotic synergies that may enhance antioxidant metabolite synthesis in wild samples [60]. This could explain why BL exhibits the lowest levels of essential amino acids (except lysine) and functional components, as well as the weakest antioxidant capacity among all four sample groups.

Correlation analysis revealed significant positive associations between antioxidant activity and functional components, suggesting that accumulation of these compounds may enhance antioxidant capacity. TIC showed reproducible peak patterns across samples, validating the reliability of the LC-MS/MS analysis. A total of 82 amino acid metabolites were identified, categorized into amino acids and metabolomics (78.6%), organic acids and their derivatives (20.21%), and others (3.19%). The higher content of organic acids and their derivatives in the fermented product BL compared to those in wild *O. sinensis* (NQ, XJ) and artificially cultivated *O. sinensis* (RG) is likely closely associated with its specific fermentation process. During fermentation, the artificially regulated environment (such as temperature, PH value, and oxygen content) can more precisely meet the metabolic demands of the strain, facilitating the efficient expression of enzymes related to organic acid synthesis [61,62]. In contrast, wild and artificially cultivated *O. sinensis* are subject to fluctuations in the natural environment or limitations in cultivation conditions, which may prevent their enzyme activities and metabolic pathways from reaching optimal states. Meanwhile, the strain used in BL, after screening and optimization, may possess a stronger capability for organic acid biosynthesis, and its metabolic network tends to accumulate such compounds [63,64]. In comparison, the metabolic direction of wild strains focuses more on adapting to natural survival competition, and the strain of RG may not have specifically enhanced the organic acid synthesis pathway during the domestication process. These factors collectively contribute to the significant advantage of the fermented product BL in the accumulation of organic acids and their derivatives [65,66,67].

Multivariate statistical analyses revealed distinct metabolic profiles among samples. PCA demonstrated tight clustering of NQ and XJ, whereas RG and BL formed separate clusters, indicating that geographic origin exerts a stronger influence on metabolic features than individual variation. HCA further corroborated the metabolic distinctions identified by PCA and OPLS-DA. The HCA heatmap revealed a clear separation between wild *O. sinensis* (NQ and XJ) and those from cultivated (RG) and substitute (BL) groups, closely reflecting the clustering trends observed in both the unsupervised PCA and supervised OPLS-DA models. Remarkably, NQ, XJ and RG formed a tightly clustered group enriched in aromatic and branched-chain amino acids, while BL exhibited the most divergent metabolic profile, consistent with its distinct positioning in multivariate analyses.

Wild *O. sinensis*, especially XJ, contained significantly higher levels of essential amino acids such as phenylalanine, lysine, methionine, leucine valine. Among the four samples, BL exhibits the lowest levels of all essential amino acids except for lysine. These amino acids are vital for human health. Besides differences in absolute content, metabolic networks and their physiological implications varied among groups. For example, lysine, essential for collagen synthesis, supports tissue repair and wound healing, which aligns with traditional uses of *O. sinensis* in treating chronic inflammatory diseases like arthritis [68,69]. Methionine, a precursor of glutathione, plays key roles in redox homeostasis and epigenetic regulation through methylation [70,71]. Threonine maintains intestinal mucosal integrity and promotes mucin synthesis, contributing to gut health under normal and pathological conditions [72,73]. Elevated lysine, methionine, and threonine levels in *NQ* and *XJ* suggest potential for developing antiviral, hepatoprotective, and gut-targeted therapeutics, respectively. These findings highlight the importance of essential amino acid profiling as a core metric in *O. sinensis* quality evaluation.

Following multivariate statistical analysis, five common DAAMs were identified. Subsequently, we carried out a comprehensive exploration of the content disparities of these metabolites (1,3-Dimethyluric-Acid, Trimethylamine-N-Oxide, 3-Iodo-L-Tyrosine, N-Propionylglycine, and anserine) across four components, namely NQ, XJ, RG, and BL. 1,3-Dimethyluric acid, an organic acid derivative with direct antioxidant activity, may serve as a key contributor to the overall antioxidant capacity of *O. sinensis*. Given that *O. sinensis* is well-documented for its ability to mitigate oxidative damage, 1,3-dimethyluric acid could act as an intrinsic functional component within it by scavenging reactive oxygen species (ROS) and inhibiting lipid peroxidation, thereby helping maintain redox balance in *O. sinensis*-associated biological systems. This aligns with its demonstrated role in other models, such as suppressing lipid peroxidation in human erythrocyte membranes in in vitro studies [74]. N-Propionylglycine, classified as an organic acid derivative and an intermediate in glycine metabolism, may indirectly support the antioxidant processes of *O. sinensis*. *O. sinensis* is known to regulate the body’s endogenous antioxidant defense system, and N-propionylglycine could participate in this regulation by influencing the conversion of glycine to glutathione (a critical endogenous antioxidant). If *O. sinensis* modulates the content of N-propionylglycine, it may alter the availability of glycine for glutathione synthesis—thereby adjusting the overall antioxidant capacity of the fungus or the organisms it interacts with. For instance, in specific physiological states, fluctuations in N-propionylglycine levels might signal changes in the efficiency of glycine-related antioxidant pathways within *O. sinensis* ecosystems [75]. Trimethylamine-N-oxide, a nitrogen-containing organic compound, has a complex association with oxidative stress in the context of *O. sinensis*. While trimethylamine-N-oxide does not directly participate in antioxidant synthesis, its content variations may reflect the nitrogen metabolism balance of *O. sinensis* and the fungus’s adaptation to oxidative stress. *O. sinensis* is capable of thriving under diverse environmental stressors, and trimethylamine-N-oxide could potentially act as a “stress indicator” for this fungus. For example, under oxidative stress conditions such as extreme temperatures or nutrient limitation, the levels of trimethylamine-N-oxide in *O. sinensis* might shift. This shift links to the fungus’s redox regulation mechanisms. This phenomenon echoes trimethylamine-N-oxide’s reported role in protecting against oxidative-stress-mediated functional impairments in some cell models, and this protective role may be conserved in *O. sinensis*-related biological processes [76]. 3-Iodo-L-tyrosine, an intermediate in amino acid metabolism (and an iodinated derivative of tyrosine), may exert indirect effects on the antioxidant system of *O. sinensis*. As *O. sinensis* relies on robust amino acid metabolism to sustain its growth and functional properties, 3-iodo-L-tyrosine could modulate the fungus’s tyrosine metabolic pathway, which is an axis interconnected with antioxidant-related processes. Changes in its content might disrupt or enhance the synthesis of tyrosine-derived metabolites (e.g., certain phenols or enzymes) that contribute to *O. sinensis*’s antioxidant defense, thereby indirectly shaping the fungus’s ability to resist oxidative damage [77]. Anserine, a dipeptide with direct antioxidant functions, may potentially serve as a functional component that contributes to enhancing the antioxidant properties of *O. sinensis*. *O. sinensis* is widely recognized for its ability to help reduce oxidative stress in host organisms [78], and anserine’s capacity to scavenge free radicals and chelate metal ions might complement other antioxidants (e.g., phenols, polysaccharides) in *O. sinensis*, which could aid in forming a synergistic defense network. This phenomenon may mirror anserine’s independent role observed in some other biological systems, where it has been found to protect cells from free radical-induced harm, and such a role might also extend to supporting *O. sinensis*’s intrinsic antioxidant value [79,80].

As an organic acid derivative with direct antioxidant activity, 1,3-Dimethyluric-Acid exhibits a content gradient where XJ has the highest level, followed by NQ, then RG, and BL has the lowest. It also shows a positive correlation with antioxidant indices and functional components. This implies that samples with higher content of this metabolite (e.g., XJ and NQ) possess stronger antioxidant capacity, which is consistent with the characteristic of wild *O. sinensis* (XJ and NQ) having superior inherent antioxidant performance. In contrast, the four DAAMs, namely trimethylamine-N-oxide, 3-iodo-L-tyrosine, N-propionylglycine, and anserine, exhibit the highest contents in BL. For example, trimethylamine-N-oxide, 3-iodo-L-tyrosine, N-propionylglycine, and anserine all reach their peak levels in BL, and some are even undetectable in wild samples. Specifically, trimethylamine-N-oxide content is 0 in NQ, 3-iodo-L-tyrosine and N-propionylglycine contents are 0 in XJ, and anserine content is 0 in both NQ and RG. Importantly, these four metabolites all display a negative correlation with antioxidant indices and functional components. This negative correlation, combined with their highest content in BL, further confirms the previous finding that BL has the lowest antioxidant capacity among all sample groups, which is consistent with the overall antioxidant capacity gradient where wild *O. sinensis* (XJ and NQ) shows higher antioxidant capacity than RG, and BL ranks the lowest. Additionally, this pattern suggests that the high accumulation of these metabolites may either inhibit the antioxidant capacity of the samples or reflect a compensatory state of the antioxidant system. In contrast, the low content or absence of these metabolites in wild samples ensures the efficient operation of antioxidant-related metabolic pathways, thereby maintaining strong antioxidant capacity. Further KEGG enrichment analysis revealed that DAAMs were enriched in tyrosine metabolism and general metabolic pathways. Tyrosine metabolism, a central route in aromatic amino acid metabolism, intersects with tryptophan and phenylalanine pathways, and through intermediates such as DOPA, contributes to free radical scavenging and antioxidant activity.

## 5. Conclusions

This study presents a comparative investigation of amino acid metabolism and antioxidant capacity among wild *O. sinensis* from NQ and XJ, RG, and BL, utilizing targeted LC-MS/MS metabolomics and antioxidant assays. A total of 82 amino acids and their derivatives were identified, revealing distinct metabolic divergence across the four groups. Wild samples exhibited distinct metabolic characteristics, with XJ displaying significantly higher levels of essential amino acids and NQ showing superior antioxidant activity. The latter was positively correlated with functional components such as TPS, TPE, and TF. Specific amino acid derivatives, including 1,3-Dimethyluric-Acid and Trimethylamine-N-Oxide, showed notable associations with antioxidant performance. Key metabolic pathways, such as arginine and proline metabolism, aromatic amino acid biosynthesis, tryptophan metabolism, and tyrosine metabolism, may potentially serve as the basis for quality differences and antioxidant mechanisms.

Future research could focus on elucidating the molecular mechanisms by which key amino acid derivatives modulate antioxidant activity, particularly 1,3-Dimethyluric-Acid. Additionally, exploring strategies to enhance the functional properties of cultivated *O. sinensis* and substitutes by targeting tyrosine metabolism and other relevant pathways may hold promise for industrial applications. Further studies optimizing cultivation conditions based on metabolic insights could facilitate improved accumulation of bioactive components. Integrating amino acid metabolic profiles into quality evaluation systems may also provide a more robust framework for the standardization and development of *O. sinensis* resources.

## Data Availability

The original contributions presented in this study are included in the article/Supplementary Material. Further inquiries can be directed to the corresponding authors.

## Supplementary Materials

The following supporting information can be downloaded at: https://www.mdpi.com/article/10.3390/jof11100711/s1, Table S1: Detailed information of standard products; Table S2: Equations for quantitative determination of 94 amino acid metabolites.
